# Comment on Inghilleri, G.; Franchini, M. Hypophosphatemia in Patients Receiving Intravenous Iron Supplementation for Iron-Deficiency Anemia: A Narrative Review. *J. Clin. Med.* 2026, *15*, 4748

**DOI:** 10.3390/jcm15176645

**Published:** 2026-08-28

**Authors:** Ceren Çevik, Nur Düzen Oflas, Kamil Konur, Gökhan Tazegül, Nurhayat Özkan Sevencan, Muhammet Özbilen

**Affiliations:** 1 Department of Internal Medicine, Karabük University Education and Research Hospital, 78200 Karabük, Türkiye; drcerencevik@gmail.com (C.Ç.); dr_nurhayat@hotmail.com (N.Ö.S.); 2 Department of Internal Medicine, Faculty of Medicine, Van Yüzüncü Yil University, 54100 Van, Türkiye; dr.nurdzn@hotmail.com; 3 Department of Internal Medicine, Faculty of Medicine, Recep Tayyip Erdogan University, 53020 Rize, Türkiye; 4 Department of Internal Medicine, Faculty of Medicine, Marmara University, 34854 Istanbul, Türkiye; drgtazegul@gamil.com; 5 Iron Deficiency Clinic, Department of Internal Medicine, Ordu University Training and Research Hospital, 52200 Ordu, Türkiye; drozbilen@gmail.com

We read with great interest the narrative review by Inghilleri and Franchini about hypophosphatemia in patients receiving intravenous iron supplementation for iron-deficiency anemia [1]. The authors review thoroughly the pathophysiology, risk factors, and clinical implications of hypophosphatemia associated with intravenous iron therapy mediated by FGF23. Their focus on monitoring of serum phosphate in high-risk patients is a welcome addition to clinical practice. We would like to discuss three points that we think would further enhance the clinical applicability of the review.

First, the review’s terminology regarding the specificity of hypophosphatemia to ferric carboxymaltose (FCM) warrants closer examination. Hypophosphatemia is repeatedly framed as an effect specific to this formulation. The review states most explicitly that the cascade is thought to be unique to FCM, and that although ferumoxytol and ferric derisomaltose (FDI) may cause hypophosphatemia at lower frequency and severity, these agents do not induce the so-called 6H syndrome [1]. This framing conflates two distinct claims: that isolated hypophosphatemia is FCM-specific, and that the 6H syndrome as a whole is FCM-specific. The available evidence supports neither as a binary distinction. Studies cited elsewhere in the review demonstrate that iron polymaltose [2,3] and saccharated ferric oxide [4] can each induce the same core pathophysiological sequence. This sequence comprises elevated intact FGF23, suppressed 1,25-dihydroxyvitamin D, renal phosphate wasting, and, in reported cases, symptomatic osteomalacia. These are precisely the components that define the 6H syndrome. Their occurrence with non-FCM formulations therefore indicates that the syndrome itself, and not merely isolated hypophosphatemia, arises along a graded continuum rather than being confined to a single agent.

Consistent with this view, randomized trials of FDI and iron sucrose have reported hypophosphatemia incidences of 3.9% and 2.3%, respectively [5]. A network meta-analysis directly comparing intravenous iron formulations similarly found a significant but graded, formulation-dependent risk rather than a categorical one [6]. We therefore suggest that hypophosphatemia is more accurately characterized as a formulation-dependent effect shared, to differing degrees, across carbohydrate-stabilized intravenous iron complexes, occurring along a spectrum of frequency and severity. This interpretation is consistent with a recent multidisciplinary consensus, which links the risk most prominently to FCM and, to a lesser extent, to several other formulations [7]. This framing is not intended to minimize the comparatively higher risk associated with FCM, which is well supported by the data summarized in the review. However, explicitly acknowledging hypophosphatemia as a shared, mechanism-based effect that is not confined to FCM would help prevent false reassurance regarding the safety of alternative formulations in at-risk patients. This distinction carries practical relevance, given that the review’s monitoring recommendations apply almost exclusively to FCM. Consequently, in the management of hypophosphatemia and the synthesis of associated algorithms, the phrase “IV iron-induced hypophosphatemia” would be more comprehensive than “FCM-induced hypophosphatemia.”

Second, the review’s management algorithm merits further discussion regarding the treatment of severe hypophosphatemia specifically. The review comprehensively describes the risk factors for hypophosphatemia. It also details the serious complications of its severe form, including cardiac arrhythmias, neurological manifestations, respiratory muscle dysfunction, and osteomalacia. In its management section, routine prophylactic phosphate supplementation is discouraged, on the grounds that it may exacerbate FGF23-mediated renal phosphaturia. Observation for mild or asymptomatic hypophosphatemia, avoidance of further FCM administration, and management of secondary hyperparathyroidism are emphasized as the principal therapeutic strategies. Importantly, however, this rationale primarily pertains to prophylactic use. It should not be conflated with the management of severe or symptomatic hypophosphatemia, for which an individualized approach based on disease severity, clinical manifestations, and underlying etiology is more appropriate [8].

In this regard, the review’s algorithm recommends continued observation even for severe hypophosphatemia, with intravenous phosphate replacement considered only if symptoms are present and phosphate levels are not increasing. We would question an observation-only default in this specific setting. Although reported rates vary with the population studied, formulation, phosphate threshold, dose, repeated exposure, and timing of measurement, severe hypophosphatemia is not rare with FCM: the review itself reports severe hypophosphatemia (serum phosphate ≤ 1.0 mg/dL) in 11.3% of FCM-treated patients in the pooled PHOSPHARE trials, and real-world rates of severe or profound hypophosphatemia of 7–13% [1]. A default of observation is therefore difficult to justify in this setting. Precisely because acute severe hypophosphatemia can impair cardiac and respiratory function and, as the review itself notes, may be life-threatening, it warrants consideration of active management, individualized according to symptom burden, the depth of hypophosphatemia, and institutional protocols. A plan of reevaluating phosphate levels only after 3–4 weeks is difficult to reconcile with the follow-up intensity that a potentially life-threatening biochemical derangement requires. Short-interval monitoring, together with consideration of inpatient management under institutional phosphate replacement protocols, would be more appropriate for this subgroup.

This position aligns with recent expert consensus statements, which place greater emphasis on active management. Mild-to-moderate FCM-associated hypophosphatemia should be observed according to the 2025 consensus. Management of severe hypophosphatemia should be based on institutional phosphate replacement protocols with consideration for re-evaluation of the need for continued FCM therapy if hypophosphatemia persists [9]. Similarly, the 2026 NATA consensus provides a practical algorithm with treatment of symptomatic or severe hypophosphatemia, and switch to alternative formulations as appropriate [7]. Taken together, these recommendations underscore the importance of distinguishing prophylactic phosphate supplementation from the therapeutic management of severe or symptomatic disease.

In addition, we wish to highlight an unresolved evidence gap concerning the recommendation to switch to an alternative formulation once hypophosphatemia develops. This recommendation rests largely on comparative incidence data from treatment-naive populations, which may not encompass all available observational evidence. The review itself, however, identifies a history of preexisting hypophosphatemia as a risk factor for future hypophosphatemia. We are not aware of prospective studies specifically evaluating patients switched to another intravenous iron formulation after developing FCM-associated hypophosphatemia. The comparative safety data that motivate switching may therefore not be fully transferable to this particular population. Until such data become available, the appropriateness of switching to an alternative intravenous iron formulation after the onset of hypophosphatemia remains uncertain, and we suggest that this uncertainty be made explicit in any management algorithm.

Third, we noted a related ambiguity in the terminology used for “vitamin D supplementation” in the management recommendations. The review correctly describes how FGF23 suppresses 1α-hydroxylase; FGF23 concurrently accelerates catabolism of the active metabolite by inducing 24-hydroxylase, so that reduced synthesis and increased inactivation together lower 1,25-dihydroxyvitamin D rather than depleting 25-hydroxyvitamin D substrate stores [10,11]. This dissociation is reflected in the biochemical profile regarded as typical of intravenous iron-induced hypophosphatemia, in which 25-hydroxyvitamin D is normal or only mildly reduced while 1,25-dihydroxyvitamin D is markedly reduced [10]. However, the recommendation to consider vitamin D supplementation for mitigating secondary hyperparathyroidism does not distinguish between native vitamin D (cholecalciferol/ergocalciferol) and active vitamin D analogues (calcitriol/alfacalcidol), although the two address different problems. Because the underlying defect is partly enzymatic rather than solely substrate-limited, native vitamin D repletion alone may be insufficient to restore 1,25-dihydroxyvitamin D synthesis while 1α-hydroxylase activity remains suppressed. Consistent with this substrate-independent mechanism, several studies have found that baseline vitamin D status does not independently predict the risk of hypophosphatemia following FCM administration [12,13], and whether vitamin D given before FCM reduces hypophosphatemia or fracture risk remains unknown [10].

This does not, however, exclude a therapeutic role for correcting coexisting native vitamin D deficiency, which remains relevant on separate grounds: secondary hyperparathyroidism has been considered an unlikely cause of intravenous iron-induced osteomalacia unless associated with severe vitamin D deficiency [10], and native vitamin D was administered to all 13 patients in the largest published cohort, 6 of whom additionally received an active analogue [11]. Active analogues, by contrast, are directed at the enzymatic block itself, and this is reflected in reported practice. In a structured literature search of 77 cases, activated vitamin D was among the treatments used in 21 patients, more often than native vitamin D (*n* = 9), although outcomes were variably and inconsistently reported; on mechanistic grounds the same review proposes mitigation of secondary hyperparathyroidism with activated vitamin D as a rational treatment approach that has been used successfully in some patients [10]. In the largest cohort (*n* = 13), serum 1,25-dihydroxyvitamin D rose significantly during treatment while 25-hydroxyvitamin D and PTH did not; because FCM was also stopped or switched in 12 of these patients, this cannot be attributed to vitamin D supplementation alone. The exception is instructive: in the one patient in whom FCM had to be continued, supportive therapy with alfacalcidol, phosphate and calcium reduced bone-specific alkaline phosphatase and improved bone mineral density despite persistent FGF23 excess, until a plateau prompted off-label anti-FGF23 therapy [11]. Normalization of calcium and phosphate after calcitriol initiation has also been described in an individual report [14]. Active vitamin D therefore appears adjunctive rather than curative while FGF23 remains elevated, and warrants monitoring of serum and urinary calcium. Accordingly, active vitamin D analogues may be considered in selected patients with persistent hypophosphatemia, hypocalcemia, or secondary hyperparathyroidism; native vitamin D deficiency, when present, should also be corrected. Specifying the intended form of vitamin D, and the distinct rationale for each, would help clinicians match therapy to the underlying hormonal deficit.

We commend the authors for a thorough and clinically relevant review of this increasingly recognized adverse effect, and we hope these observations contribute constructively to the ongoing clinical discussion around intravenous iron-associated hypophosphatemia.

## Data Availability

No new data were created or analyzed in this study. Data sharing is not applicable to this article.
